# Probing Mechanistic Insights into Highly Efficient Lithium Storage of C_60_ Fullerene Enabled via Three‐Electron‐Redox Chemistry

**DOI:** 10.1002/advs.202101759

**Published:** 2021-07-11

**Authors:** Haifa Qiu, Jing Wan, Junxian Zhang, Xin Wang, Nianji Zhang, Rouxi Chen, Yu Xia, Li Huang, Hsing‐Lin Wang

**Affiliations:** ^1^ Shenzhen Key Laboratory of Solid State Batteries Southern University of Science and Technology Shenzhen 518055 China; ^2^ Department of Materials Science and Engineering Southern University of Science and Technology Shenzhen 518055 China; ^3^ Department of Physics Southern University of Science and Technology Shenzhen 518055 China; ^4^ Academy for Advanced Interdisciplinary Studies Southern University of Science and Technology Shenzhen 518055 China; ^5^ Guangdong Provincial Key Laboratory of Energy Materials for Electric Power Southern University of Science and Technology Shenzhen 518055 China

**Keywords:** C_60_ fullerene, lithium storage, organic cathode, phase transition, three‐electron‐redox

## Abstract

Renewable organic cathodes with abundant elements show promise for sustainable rechargeable batteries. Herein, for the first time, utilizing C_60_ fullerene as organic cathode for room‐temperature lithium‐ion battery is reported. The C_60_ cathode shows robust electrochemical performance preferably in ether‐based electrolyte. It delivers discharge capacity up to 120 mAh g^−1^ and specific energy exceeding 200 Wh kg^−1^ with high initial Coulombic efficiency of 91%. The as‐fabricated battery holds a capacity of 90 mAh g^−1^ after 50 cycles and showcases remarkable rate performance with 77 mAh g^−1^ retained at 500 mA g^−1^. Noteworthily, three couples of unusual flat voltage plateaus recur at ≈2.4, 1.7, and 1.5 V, respectively. Diffusion‐dominated three‐electron‐redox reactions are revealed by cyclic voltammogram and plateau capacities. Intriguingly, it is for the first time unveiled by in situ X‐ray diffraction (XRD) that the C_60_ cathode underwent three reversible phase transitions during lithiation/delithiation process, except for the initial discharge when irreversible polymerization in between C_60_ nanoclusters existed as suggested by the characteristic irreversible peak shifts in both in situ XRD pattern and in situ Raman spectra. Cs‐corrected transmission electron microscope corroborated these phase evolutions. Importantly, delithiation potentials derived from density‐functional‐theory simulation based on proposed phase structures qualitatively consists with experimental ones.

## Graphical abstract

Renewable C_60_ fullerene is for the first time applied as organic cathode for room‐temperature Li‐ion battery, showcasing unusual electrochemical performance. Unlike previously reported cases, the voltage profiles of C_60_ fullerene in this study unusually exhibit three flat plateaus, corresponding to three‐electron‐transfer. Further, three reversible phase transitions are revealed by in situ XRD, ex situ TEM, and DFT calculations.

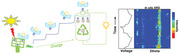

## 1 Introduction

Li‐ion batteries (LIBs) have become prevailing mobile power sources and play indispensable roles in revolutionizing modern society owing to their high energy density merit, with applications spanning consuming electronics, lightweight smart devices, electrical transportation vehicles and stationary energy storage systems.^[^ ^1^, ^2^, ^3^, ^4^, ^5^, ^6^, ^7^ ^]^ However, one of the challenges impeding the advancement of LIBs is the unsustainability of current commercial cathode materials, including LiMn_2_O_4_, LiFePO_4_, and Li(Ni, Co, Mn)O_2_, on account of limited reserves of lithium and transition metals, the eco‐malignity of cobalt, high cost, problematic recyclability and adverse environmental impacts.^[^ ^9^, ^10^ ^]^ In recent years, the metal‐free organic (MFO) materials composed of abundant elements (C, H, O, N, S) have emerged as novel cathode materials for rechargeable batteries due to their renewability, structural diversity and componential tunability over the aforementioned conventional cathode materials.^[^ ^11^, ^12^, ^13^, ^14^, ^15^, ^16^, ^17^ ^]^ These MFO cathode materials mainly fall into three types: *n*‐type, *p*‐type, and bipolar depending on the accessible charge‐state of the electroactive sites within.^[^ ^12^, ^18^ ^]^ In the charging process, the *p*‐type materials, such as polyphenylamine, coronene, and its analogues, can be oxidized accompanied by anion (such as PF_6_⁻ and ClO_4_⁻) intercalation for dual‐ion batteries (DIBs).^[^ ^19^, ^20^ ^]^ In the discharging process, *n*‐type materials such as cyclohexanehexone, calix[4]quinone, calix[6]quinone and polydopamine can be reduced to anions concurring with Li‐ion intercalation for LIB.^[^ ^21^, ^22^, ^23^, ^24^ ^]^ Noteworthily, some *n*‐type MFO cathode materials for LIBs are molecular crystals with multi‐carbonyls and they share a common trait of multi‐electron‐redox chemistry caused by the reactions of multi‐carbonyls with lithium ions, which renders them higher capacity than those *p*‐type ones with less electron transfer for DIBs. Intriguingly, fullerenes, C_60_ being the most common form, discovered by Kroto et al. in 1985 as an unusual form of carbon allotrope with a Buckyball cage‐like molecular structure comprising twenty pentagonal and twelve hexagonal fused rings of single or double bonded carbon atoms, are known as bipolar molecular crystals and peculiarly display multi‐electron‐redox behavior.^[^ ^25^, ^26^, ^27^ ^]^ Although fullerenes have been studied as electrode materials for rechargeable batteries including redox flow batteries, little work has been reported of their use as cathode material for LIB.^[^ ^30^, ^31^, ^32^, ^33^ ^]^ Previously reported DIB and magnesium‐ion battery (MIB) with C_60_ fullerene as cathode material delivered reversible capacity below 50 mAh g^−1^ and exhibited less than ideal cycling stability.^[^ ^13^, ^34^, ^35^ ^]^ Moreover, the role and battery chemistry of multi‐electron‐redox in these fullerene batteries remain elusive.^[^ ^36^ ^]^ In this work, using C_60_ as a prototype of fullerenes, we have successfully fabricated the proof‐of‐concept rechargeable LIB with C_60_ fullerene serving as cathode material that can operate in room temperature and revealed the underlying mechanisms through a series of in situ/ex situ characterizations along with DFT computation. The C_60_ cathode exhibits prominent performance for LIB with high initial Coulombic efficiency (CE) and a capacity over twice as those of the fullerenes reported for DIB and MIB, which is also superior among organic cathode materials without carbonyls including most *p*‐type organic cathode materials for DIB and even comparable to some *n*‐type materials for LIB.^[^ ^11^, ^19^, ^21^, ^35^, ^37^ ^]^ Our work could render further insight into the fullerene battery chemistry and can be enlightening for their applications in rechargeable batteries and beyond.

## 2 Results and Discussions

**Figure 1:**
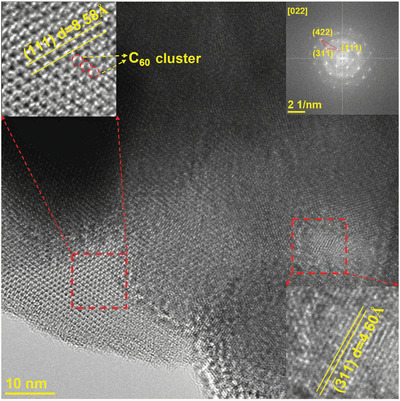
a) High resolution TEM image of pristine C_60_. The upper left and the lower right insets are magnified views, while the upper right inset is corresponding FFT pattern.

The pristine C_60_ in our study is brownish powder that was used as received without further treatment. Its morphology at microscale is characteristic of bulk secondary particles closely stacked from submicro‐sized flakes as revealed by Scanning electron microscopy (SEM) in Figure S1, Supporting Information. As shown in the X‐ray diffraction (XRD) pattern in Figure S2, Supporting Information, three major peaks located at ≈10.8°, 17.7°, and 20.8° are ascribed to (111), (220), and (311) crystal planes in face‐centered cubic (FCC) structure belonging to the Fm$\bar{3}$ space group, indicative of single crystalline trait of C_60_ and consistent with previously reported work.^[^ ^30^ ^]^ The distinct lattice fringes and their spacings in the high‐resolution TEM image, along with the FFT pattern in the upper right inset, correspond well to the XRD results, as shown in **Figure** **1**. Besides, C_60_ nanoclusters were also distinguished as shown in the upper left inset.

**Figure 2:**
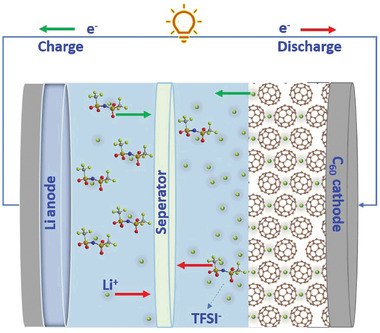
Schematic illustration of the construction of LIB with C_60_ as cathode and the charge/discharge process.

**Figure 3:**
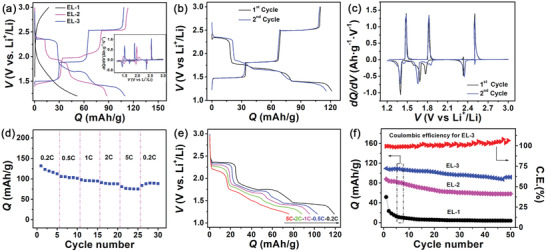
Electrochemical behavior and performance of C_60_ as cathode material in half cells measured at room temperature: a) the GDC voltage profiles for different electrolytes at 1 C current rate, inset are corresponding *dQ/dV* curves, here *V* stands for voltage, b) voltage profiles of initial 2 GDC cycles for EL‐3 electrolyte, c) differential capacitance curves derived from voltage profiles of (b), d) rate capability of C_60_ cathode at various current rates, e) discharge voltage profiles for the third cycles at various current rates, f) cycling performance of C_60_ cathode in varied electrolytes, here C.E. denotes Coulombic efficiency.

The pure electrochemistry of C_60_ fullerene has been well established since its discovery.^[^ ^27^ ^]^ However, little attention has been concentrated on its electrochemical behavior in batteries and the knowledge pertaining to battery chemistry remains limited.^[^ ^30^, ^35^, ^36^, ^38^, ^39^ ^]^ Therefore, a main objective of this work is to investigate the electrochemical behavior of C_60_ as LIB cathode material. The schematic architecture of C_60_ LIB and its operating principle was illustrated in **Figure** **2**. We began by studying the dependence of electrochemical behavior in half cells. **Figure** **3**a exhibit typical galvanic discharge‐charge (GDC) voltage profiles at 1 C (refers to 100 mA g^−1^) of current rate after 2 cycles at 0.2 C in three different electrolytes. For EL‐1, the electrolyte of 1 M LiPF_6_ dissolved in mixed solvent of ethylene carbonate, diethyl carbonate, and dimethyl carbonate (1:1:1 in volume), the discharge‐charge voltage profiles of C_60_ displays no voltage plateaus, similar to previous work.^[^ ^30^ ^]^ Both the CE (here defined as the ratio of charge capacity to discharge counterpart) of 33.5% and reversible capacity of less than 20 mAh g^−1^ are very low. This could be rationalized by the deterioration of C_60_ from the irreversible parasitic reactions with electrolyte in the first several cycles, which contributes to the interfacial capacitance. Further details are provided in Figure S3a, Supporting Information. On the contrary, multiple distinct voltage plateaus are observed in discharge‐charge voltage profiles for EL‐2, the electrolyte of 1 M LiClO_4_ in the mixture of dimethyl ether (DME) and dioxolane (DOL) (1:1 in volume) or EL‐3, the electrolyte of 1 M LiTFSI in DME and DOL (1:1 in volume). The three major voltage plateaus are presented altogether for both the discharge and charge process, including the high plateaus at ≈2.5 V, the middle plateaus between 1.6 and 2.1 V, and the low plateaus near 1.5 V. The GDC CE of C_60_ for EL‐2 and EL‐3 are 128.8%, and 98.7%, respectively. The much higher CE near 100% and much higher discharge capacity of ≈107 mAh g^−1^ demonstrates excellent electrochemical reversibility and redox activity of the C_60_ cathode at such voltage range at 1 C for the EL‐3 electrolyte, whereas the abnormally high CE for EL‐2 may result from shuttle effect of dissolved active materials, presumably driven by side chemical and/or electrochemical reactions of the lithiated C_60_ with ClO_4_⁻.^[^ ^40^, ^41^ ^]^ Underlying redox difference is reflected in corresponding *dQ/dV* curves in the inset of Figure 3a, where no redox peaks are observed and the curves are almost flat for EL‐1 while the redox peaks are quite strong for EL‐2 and EL‐3. Further, half cells with the C_60_ cathode were tested in 0.1 M LiPF_6_ in DME. Surprisingly, we find that the voltage profiles also exhibit distinct plateaus as displayed in Figure S3b, Supporting Information. As observed in the CV curves in Figure S3c, Supporting Information, below 1.2 V extra reduction emerges and there are no more redox peaks for C_60_ cathode after the first two cycles, due to the irreversible strong reduction peak ≈0.7 V where irreversible intense parasitic interfacial reactions could occur with the collapse of C_60_ structure. And this leads to the eventual loss of redox activity for C_60_. All the above results manifest that, apart from the voltage range, the electrochemical redox behavior of C_60_ as cathode is strongly dependent on the electrolyte, with more influence from the solvent than from the solvate. More specifically, the DME‐based electrolyte rather than the carbonate‐based electrolyte is preferable for the redox of the C_60_ cathode in LIB.

Furthermore, the electrochemical properties were evaluated for the C_60_ cathode in half cells with the electrolyte of EL‐3. As Figure 3b shows, in the initial cycle at 0.2 C, the C_60_ serving as cathode material can deliver capacity as high as 121.2 mAh g^−1^ corresponding to an energy density above 200 Wh kg^−1^ with remarkable CE of 90.8%, which are superior to many other MFO cathode materials for LIBs including polydopamine, cyclohexanehexone, and 2,2′‐bis‐p‐benzoquinone derivatives.^[^ ^21^, ^23^, ^37^ ^]^ The charge voltage profiles of the first two GDC cycles are almost overlapped, showcasing prominent oxidation stability. It is noted that the initial charge capacity of 110.0 mAh g^−1^ approximates the theoretical value of 111.7 mAh g^−1^ calculated based on three‐electron transfer, which suggests that the charging process of C_60_ is a three‐electron oxidation reaction. In the first cycle, the discharge voltage profile displays distinct ladder‐like voltage plateaus at 2.33, 1.68, and 1.40 V, respectively, apart from the extra minor plateau at 1.77 V. While the charge counterpart displays only three yet more elegantly flat plateaus at 2.50, 1.82, and 1.48 V that corresponds to three‐stage oxidation of the lithiated C_60_. The three‐stage voltage plateaus and their values are similar to those reported ones and the similar plateau capacity close to 37 mAh g^−1^ equivalent to one electron transfer for each plateau, particularly for the charging process, further supports the electron‐by‐electron three‐electron‐oxidation within the C_60_ fullerene during charging.^[^ ^36^ ^]^ Corresponding redox peaks are shown in Figure 3c. However, the discharge voltage plateau corresponding to the reduction peak near 1.8 V is smaller in the second cycle, which implies that the initial discharge underwent an irreversible reduction reaction and this, combined with interfacial pseudocapacitance, accounts for the irreversible capacity in the initial cycle and the relatively higher initial discharge capacity.

The rate capability of the C_60_ cathode was ascertained at various current rates at room temperature, as shown in Figure 3d,e. The average discharge capacities at 0.2, 0.5, 1, 2, and 5 C are 120.4, 103.6, 95.6, 88.6, and 76.7 mAh g^−1^, respectively. The capacity retention at 5 C is 63.7% relative to 0.2 C and the rate retention capability is equivalent to or even higher than previously reported MFO materials.^[^ ^21^, ^23^, ^24^ ^]^ A comparison in electrochemical performance for various MFO materials provided in Table S1 and Figure S8, Supporting Information, shows that the performance the C60 cathode is superior compared with other MFO cathodes without carbonyls. The relatively large interstitials in between the C_60_ clusters and the global electron delocalization in the C_60_ clusters synergistically favor charge transfer and intermolecular lithium ion diffusion and contribute to the superior rate capability.^[^ ^35^, ^42^, ^43^, ^44^ ^]^ To examine the cycling stability of the C_60_ cathode, GDC tests were performed at 0.2 C current rate for initial 2 cycles followed by 1 C current rate for another 50 cycles. As Figure 3f shows, the reversible capacity that the C_60_ cathode delivered in EL‐3 electrolyte maintained at 91.9 mAh g^−1^ with a capacity retention rate of 83.9% after 50 cycles, along with an average discharge capacity of 99.1 mAh g^−1^. Even after 100 cycles, a capacity above 70 mAh g^−1^ was retained, as shown in Figure S9, Supporting Information. In contrast, the discharge capacity for EL‐2 faded quickly in the initial 20 cycles and then remained at low level ≈66 mAh g^−1^ with a final capacity decay by 33.8%. The case for EL‐1 has a rather low capacity of only 7.2 mAh g^−1^ retained and 7.4% retention rate after 50 cycles. This manifests that the cycling stability of the C_60_ cathode is also closely related to the electrolyte used, and the EL‐3 electrolyte can better support the relatively stable GDC cycling of the C_60_ cathode than the other two. The capacity fading for EL‐3 during cycling is related to the partial dissolution of lithiated active materials similar to other organic MFO materials.^[^ ^12^ ^]^ And the dissolving extent in DME for active materials in electrodes at varied states of charge during charge‐discharge process was compared through UV–Vis spectroscopy, as shown in Figures S11 and S12, Supporting Information.

**Figure 4:**
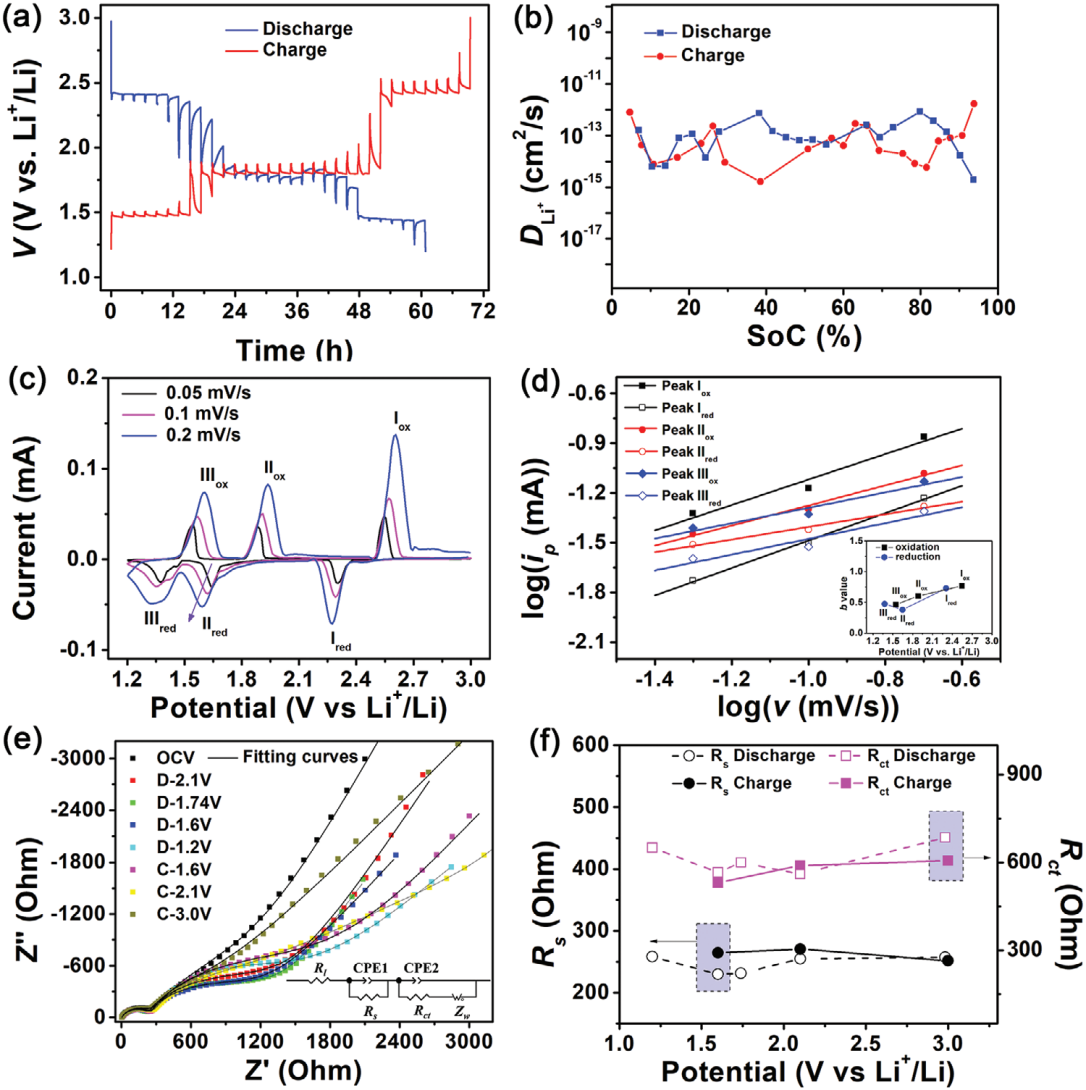
a) Voltage profiles acquired from GITT measurement for the 3rd GDC cycle, b) apparent diffusion coefficients *D* of lithium ion calculated based on (a), here the 100% SoC is defined as the lithiated state at 1.2 V, c) CV curves of C_60_ cathode at various scan rates, d) logarithm plots of peak current versus scan rate, e) Nyquist plots for C_60_ cathode obtained by EIS tests at various discharged/charged states for the 4th GDC cycle, the OCV data point near 3.0 V is categorized to the discharge part. Inset is corresponding equivalent circuit via which fitting curves are obtained, f) Evolution of resistance at different discharged or charged states extracted from fitting curves of (e).

The electrode kinetics of C_60_ cathode were studied by galvanostatic intermittent titration test (GITT), cyclic voltammetry (CV) and electrochemical impedance spectroscopy (EIS) as presented in **Figure** **4**. The apparent diffusion coefficient of lithium ion, $D_{Li^{+}}$_,_ in the C_60_ cathode was measured by GITT at 0.2 C at the third cycle, as shown in Figure 4a,b. The open circuit voltages (OCVs) for the discharge and charge plateaus derived from the voltage profiles in Figure 4a are almost identical. The average discharge OCVs are 2.41, 1.80, and 1.45 V while the average charge OCVs are 2.41, 1.80, and 1.47 V. The average $D_{Li^{+}}$ for discharge process is 1.71 × 10^−13^ cm^2^ s^−1^ similar to the value of 1.83 × 10^−13^ cm^2^ s^−1^ for charge process, implicating high reversibility in terms of lithium diffusion kinetics for the C_60_ cathode during GDC processes and comparable lithium ion diffusion kinetics relative to other MFO materials and typical cathode materials for LIBs were shown in Table S2, Supporting Information. The CV curves at various scan rates were shown in Figure 4c. Three pairs of redox peaks are observed, consistent with the second cycle in *dQ/dV* curves. The changes of current with scan rate follow the power law:

(1) $$i=av^{b}$$

The value of *b* can be obtained by plotting log(*i*) as the function of log(*v*) according to the derivation of above equation:

(2) $$\log\left(i\right)\;=\log\left(a\right)\;+b\log\left(v\right)$$

where *v* is the scan rate, *a* and *b* are tunable coefficients, with *b* as the slope of plots in Figure 4d. The electrochemical reaction is dictated by capacitive process at *b* = 1, while it indicates the capacity is dominated by solid state diffusion when *b* = 0.5.^[^ ^45^, ^46^ ^]^ The average b values for the redox peak pairs of I, II, and III are 0.749, 0.493, and 0.470, respectively, which showcases that peak pair I exhibits a mixture between the capacitive and the diffusion‐limited behavior while the other two are basically controlled by diffusion. Furthermore, the EIS curves for the C_60_ cathode at different charged or discharged states in the fourth cycle were obtained and are shown in Figure 4e. In the inset is the equivalent circuit for EIS fitting, in which the parameter *R*_l_ represents the resistance of electrolyte and the contacts of cell parts other than the active materials, *R*_s_ the solid‐electrolyte‐interface(SEI) resistance, *R*_ct_ the charge transfer resistance, and *Z*_w_ the Warburg resistance, and CPE1 and CPE2 are the constant phase elements.^[^ ^47^, ^48^, ^49^ ^]^ In a GDC cycle, *R*_s_, derived from the high‐frequency semicircle, mostly remains steady at ≈250 Ohm at various discharge and charged states, as shown in Figure 4f. However, the evolution of *R*_ct_ value, derived from the middle‐frequency semicircle, behaves more like a slight U‐type curve. It declines by 18% from 660 Ohm at the OCV state to ≈560 Ohm after discharged to 2.1 V below 1st discharge plateau, and then keeps almost stable before a bold climb to ≈651 Ohm when the cell was discharged to 1.2 V of deeply lithiated state. Over subsequent charge process, the *R*_ct_ value gradually increases to ≈607 Ohm at fully charged state. This indicates that the SEI is less sensitive to the lithium intercalation compared with the charge transfer for C_60_ cathode in this GDC cycle. It is also shown that the charge transfer can be reversibly altered by lithium intercalation. A comparison of charge transfer resistance with other MFO cathode materials in Table S3, Supporting Information, shows that the charge transfer resistance of C_60_ cathode is relatively low, suggesting decent electrode kinetics of charge transfer. Moreover, the relatively lower *R*_ct_ values at lower lithiated potentials imply that the relatively higher lithiation degree favors charge transfer kinetics.

Given the near‐theoretical reversible capacity and the redox behavior described above, the C_60_ cathode can undergo reversible three‐electron redox reactions with respect to the GDC process except for the first discharge. The reactions can be deduced as follows:

For redox pair I between 2.33 and 2.5 V,

(3) $$C_{60}+e^{-}+Li^{+}↔\mathrm{Li}C_{60}$$

For redox pair II between 1.65 and 1.82 V,

(4) $$\mathrm{Li}C_{60}+e^{-}+Li^{+}↔Li_{2}C_{60}$$

For redox pair III between 1.40 and 1.48 V,

(5) $$Li_{2}C_{60}+e^{-}+Li^{+}↔Li_{3}C_{60}$$

**Figure 5:**
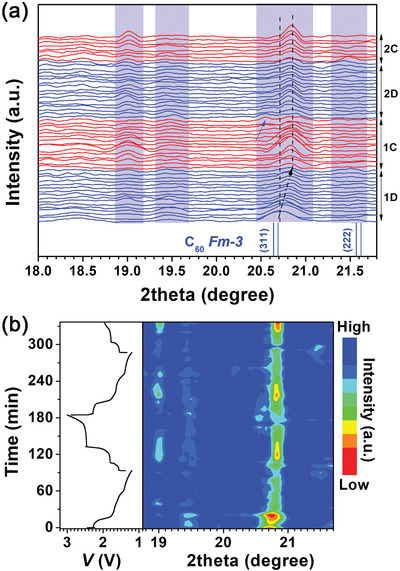
a) Evolution of XRD patterns of the (311) and new peaks of the C_60_ cathode obtained in situ during GDC processes, with 1D denoting the first discharge process, 1C the first charge process, 2D the second discharge process, and 2C the second charge process, b) corresponding 2D mapping of (a), curves on the left are the corresponding voltage profiles.

To gain insights into the structural evolution of the C_60_ cathode during the GDC processes, in situ XRD experiment was performed at 0.35 C in half cell at room temperature. The results are shown in **Figure** **5**. During the discharge‐charge process, the C_60_ cathode underwent phase transitions corresponding to three discharge voltage plateaus. For the initial discharge, as shown in Figure 5a, the (311) peak shifts positively from ≈20.70° to ≈20.85°, indicative of an interplanar contraction for the (311) crystal plane. Slight negative shift was also observed for (111) peak, as shown in the wide‐range XRD pattern in Figure S4, Supporting Information. In the meanwhile, the intensity of (311) diffraction peak decreases first and then increases to its apex when the cell was discharged to the second voltage plateau, followed by another decrease, as observed in Figure 5b. Surprisingly, one new peak 19.55° emerge during the 1st discharge voltage plateau 2.3 V, which can be ascribed to the orthorhombic phase (denoted as O1, ICSD‐75 612) belonging to Pnnm space group.^[^ ^50^ ^]^ Later when the cell was discharged to the 2nd plateau 1.6 V, another new peak appears at 19.0° and gradually intensifies, which, together with the peak near 19.5° matches monoclinic C2/m phase (denoted as M1, ICSD‐55 493).^[^ ^51^ ^]^ In the 3rd discharge plateau near 1.4 V, these peaks become weak and another peak near 21.5° appears, suggestive of another phase presumably formed of monoclinic C2/m phase (denoted as M2, ICSD‐94 237).^[^ ^36^, ^52^ ^]^ Most intriguingly, in the initial charge process, the (311) peak begin to intensify when the cell was charged to the second voltage plateau near 1.8 V while its peak position remains. When the cell was fully recharged to 3.0 V, another new peak appears at ≈20.55° (marked as blue arrow in Figure 5a) which is attributed to another orthorhombic Pnnm phase (denoted as O2, ICSD‐94 500).^[^ ^53^ ^]^ Noteworthily, the peak at ≈19° intensifies and subsides alternatingly during GDC, concurring with the peak near 20.8°, which further supports that the peak near 19.0° corresponds to monoclinic C2/m phase. Note that the peak originally from (311) plane of FCC cannot go back to its initial position at ≈20.7° in following GDC process. This irreversible peak shift could correlate with the irreversible short discharge voltage plateau near 1.77 V, which can be rationalized by partial polymerization between C_60_ clusters via intermolecular coupling.^[^ ^53^, ^54^ ^]^ This peak shift also suggests that the intermolecular coupling interaction can induce the interplanar contraction which is also irreversible. The phase structures and their evolution were further verified through ex situ transmission electron microscopy (TEM) performed for samples discharged or charged to varied cut‐off voltages (Figures S5 and S10, Supporting Information).

**Figure 6:**
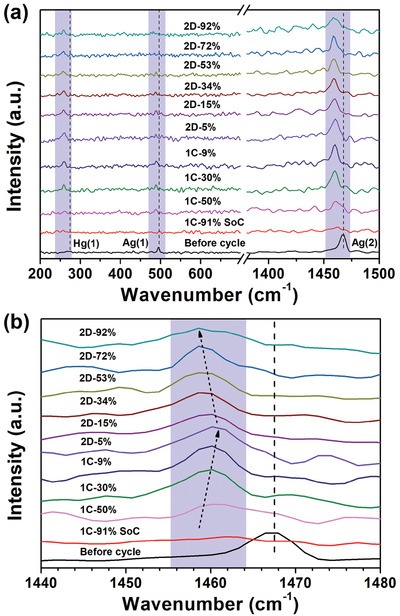
a) Evolution of Raman spectra of C_60_ cathode at various SoC acquired in situ, b) Magnified view of high‐frequency part of (a).

In situ Raman spectroscopy was employed to inspect the vibration evolution of the C_60_ cathode during GDC process. As shown in **Figure** **6**, the Raman spectra of the pristine C_60_ electrode exhibits three peaks at 272.3, 495.6, and 1468.0 cm^−1^ corresponding to the intramolecular vibration modes of “squashing” Hg(1), “breathing” Ag(1), and “pentagonal‐pinch” Ag(2), respectively.^[^ ^55^, ^56^ ^]^ The intensity of all these peaks increased during charge process of the lithium deintercalation yet decreased during discharge process of electron injection. The increased peak intensity could involve the recovered molecular symmetry with delithiation and vice versa. Moreover, the peak position also varies with the degree of lithium intercalation. For instance, the Ag(2) peak shifts toward higher frequency during charging and then shifts toward lower frequency during discharging (Figure 6b), which arises from reversible formation and decomposition of intramolecular covalent bonding induced by charge transfer.^[^ ^57^ ^]^ The irreversible downshift gaps of ≈6 cm^−1^ for all these peaks in GDC process relative to their pristine positions, similar to the aforementioned irreversible XRD peak shift (Figure 5a), suggests a reduced symmetry of the molecules.^[^ ^58^ ^]^ The peak shift of pentagonal‐pinch Ag(2) was reported as a sensitive probe for the extent of C_60_ polymerization.^[^ ^56^, ^58^ ^]^ The Ag(2) peaks during GDC locate between 1458.6 and 1460.2 cm^−1^, as exhibited in the case of photo‐ or pressure‐induced polymerized C_60_, could be attributed to the linear C_60_ clusters.^[^ ^54^, ^56^ ^]^

**Figure 7:**
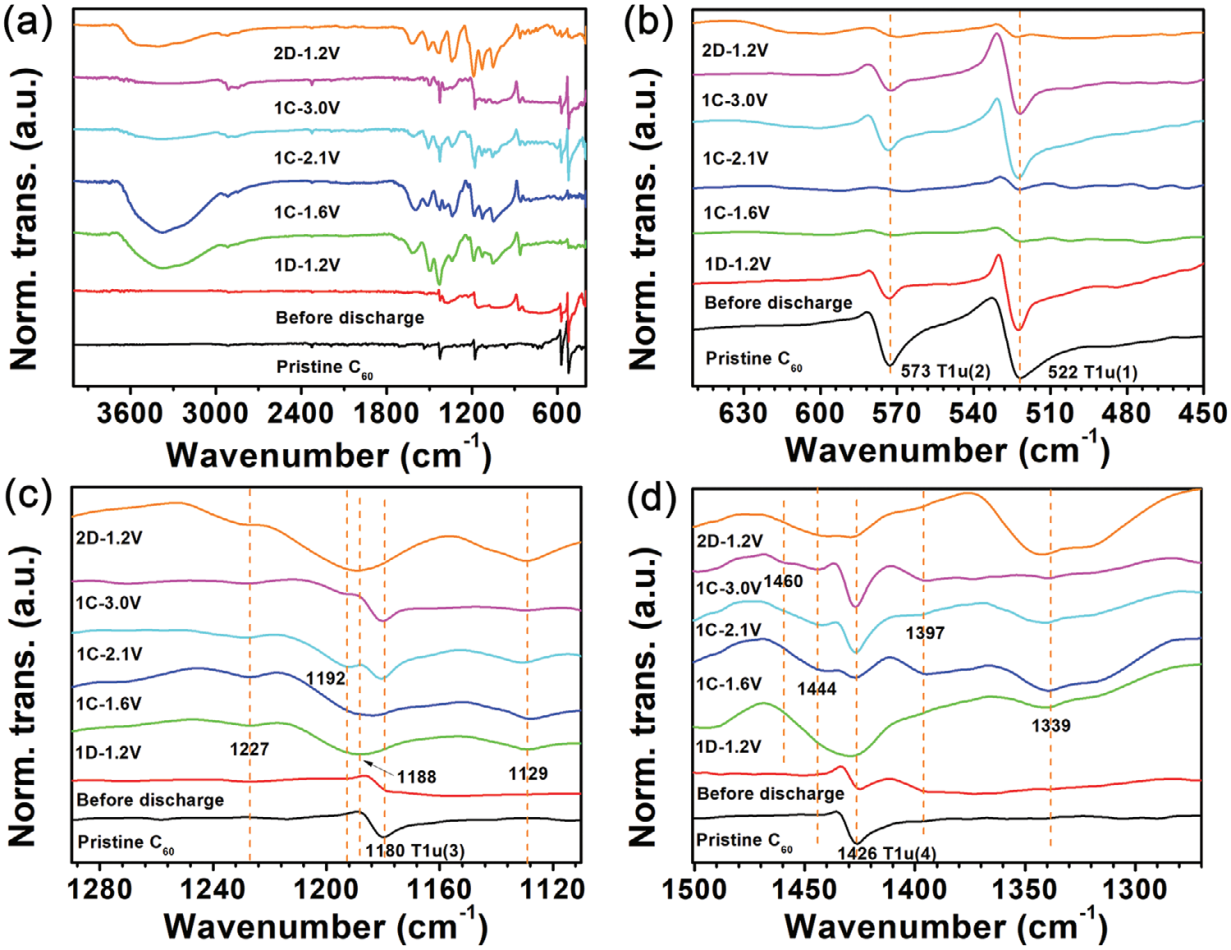
a) ATR‐IR spectra of C_60_ cathode at various discharged/charged states acquired ex situ, b–d) magnified views of (a).

Further, ex situ attenuated‐total‐reflection IR (ATR‐IR) spectroscopy was measured to detect the vibration responses of C_60_ to lithium (de)intercalation. As presented in **Figure** **7**a, the pristine C_60_ powder exhibits four major peaks located at 521.7, 572.8, 1180.2, and 1426.1 cm^−1^, which were assigned to four fundamental and symmetry‐allowed intramolecular vibration modes of T1u(1), T1u(2), T1u(3), and T1u(4), respectively.^[^ ^59^ ^]^ For charged and discharged samples, new peaks emerged, implying lowered symmetry of the C_60_ molecule.^[^ ^54^ ^]^ As shown in Figure 7b, the T1u(1) and T1u(2) peaks are first depressed after discharge and then recovered after charging. In contrast, the evolutions of high‐frequency vibrations are much more complicated due to the peak shift and emergence of new peaks. After discharge, the T1u(3) peak shifts from 1180 to 1188 cm^−1^ and becomes broadened, which implies an increased disorder among C_60_ molecules, as seen in Figure 7c. Most noteworthily, new peaks appear at ≈1227 and 1129 cm^−1^, with the latter associating with one splitting vibration mode of T1u(3).^[^ ^60^ ^]^ When the cell charged back from 1.2 to 2.1 V, the T1u(3) band underwent red shift and split, exposing another new characteristic peak at 1192 cm^−1^, related to intermolecular polymerization^[^ ^60^ ^]^ With the cell recharged to 3.0 V, the splitting peaks subside yet the T1u(3) peak resumed its original position with more sharpened shape, indicate of recovered symmetry. The reversible peak shift of T1u(3) mode could arise from the reversible fluctuation in lattice constant of the lithiated C_60_.^[^ ^61^ ^]^ As exhibited in Figure 7d, the peak shift of T1u(4) is not obvious. Similarly, T1u(4) peak also broadens up to 1465 cm^−1^ and one splitting peak appears at ≈1339 cm^−1^ come into sight.^[^ ^60^, ^62^ ^]^ As the cell charged from 1.6 to 2.1 V and to 3.0 V, new peaks emerge sequentially at 1444, 1397, and 1460 cm^−1^, consistent with those in situ IR data reported.^[^ ^62^, ^63^ ^]^ The reversible splitting of high‐frequency T1u vibrations also manifests the structural reversibility. Although the self‐polymerization between the C_60_ clusters within C_60_ cathode may lead to some irreversible capacity loss, it may also promote electron delocalization bridging the C_60_ clusters, favoring charge transfer and hence the rate capability.^[^ ^64^ ^]^

**Figure 8:**
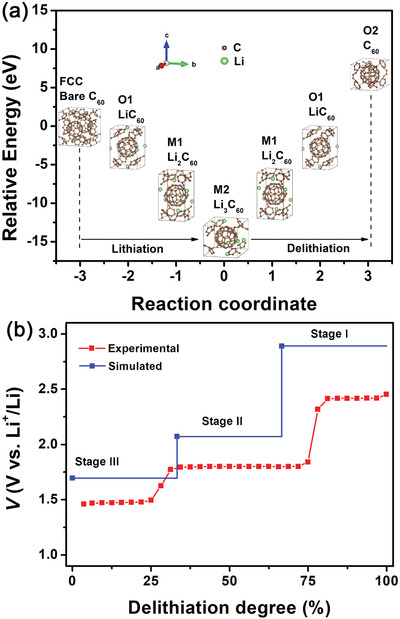
a) Schematic evolution of calculated total energy with lithiation/delithiation state for proposed phase transitions of C_60_ during initial discharge‐charge process, b) comparison between the lithium deintercalation potentials derived by DFT simulations and the quasi‐equilibrium voltage curves obtained from GITT measurements (Figure 4a). Phase structures corresponding to the data points of the simulations at the end of stages: Stage‐III M2 Li_3_C_60_, Stage‐II M1 Li_2_C_60_, Stage‐I O1 LiC_60_.

Based on the above analysis, it is deduced that the phase transitions of the C_60_ cathode during GDC were dominated by the interactions among neighboring C_60_ molecules as well as lithium intercalation. Combining in situ XRD analysis and ex situ TEM revelation with density‐functional‐theory (DFT) calculations (details are in Supporting Information), underlying mechanisms during charge and discharge were proposed, as illustrated in **Figure** **8**. Different from previous DFT studies of C_60_ based on single C_60_ molecule in cathode applications,^[^ ^65^ ^]^ plateau redox reactions and crystal phases of C_60_ with/without lithium ions involved were considered in our study to derive the delithiation potentials. For the first discharge stage, lithium ions tend to occupy the octahedral interstitial sites in FCC lattice of the C_60_ crystal for charge compensation as C_60_ molecules are negatively charged along with interplanar contraction, leading to phase transition from FCC to orthorhombic Pnnm LiC_60_ (O1 phase), accompanied by partial polymerization and volume contraction. As the lithium ions further migrate into O1 phase structure with C_60_ accepting more electrons, lithium ions can be redistributed to occupy quasi‐tetrahedral and quasi‐octahedral sites, transforming the structure to monoclinic C2/m phase (M1 phase). As more lithium ions squeeze into the M1 phase structure, redistribution of lithium ions and volume expansion can occur to accommodate more lithium ions, reshaping the structure into another C2/m phase (M2 phase)). The charging process proceeds reversibly except that another orthorhombic Pnnm (O2 phase) rather than FCC phase was formed at the end of charging. A comparison between simulated plateaus of open circuit potentials based on proposed phase structures and experimental potential profile based on GITT test is shown in Figure 8b. For simulation, it was assumed that only the oxidation of the lithiated C_60_ contributes to the charge capacity regardless of capacitance and partial polymerization. It is observed that the trend of simulated OCVs agrees qualitatively with that of the experimental OCVs, indicating that the proposed phase transition mechanism is energetically reasonable. The conformable positive potential deviation of ≈0.3 V in average can arise from disregarding the effect of solvation energy of Li^+^ and TFSI⁻.^[^ ^11^, ^66^ ^]^ It is noted that the elegant flatness of the operating voltage plateaus (Figure 3b), especially for charge part, energetically betrays the fact that the phase structures of C_60_ or the lithiated C_60_ in the midst of each plateau are stable, with little effect from intercalating lithium ion, deducing from Nernst equation and Gibbs free energy difference between the products and the reactants for a given reaction.^[^ ^11^, ^66^, ^67^ ^]^The phase transitions abruptly occur at the end of each plateau and the characteristic of multiple plateaus also distinguishes the C_60_ fullerene from most MFO multi‐electron‐redox cathode materials.

## 3 Conclusion

In summary, we used C_60_ fullerene as organic cathode for rechargeable LIB operating at room temperature. Robust electrochemical redox reactivity, much higher reversible capacity and decent cycling stability were achieved in ether‐based electrolyte relative to the carbonate‐based one. The C_60_ cathode exhibited superior electrochemical performance compared with other MFO cathodes without carbonyls, due presumably to intrinsic electron delocalization within C_60_ and beneficial lithium penetration in between C_60_ nanoclusters. Unusually, three pairs of voltage plateaus occurred sequentially at ≈2.4, 1.7, and 1.5 V in discharge‐charge voltage profiles, correlating with three‐electron‐redox chemistry demonstrated by the near‐theoretical specific total and plateau capacities. The presence of phase transitions during discharge/charge process was revealed for the first time by in situ XRD. As evidenced by the irreversible shift of (311) peak from the in situ XRD and the evolution of the peak at 1460 cm^−1^ in in situ Raman spectra, partially irreversible polymerization may be responsible for the largely irreversible minor plateau near 1.8 V. The phase evolution and irreversible structural transition was also verified by ex situ TEM.

Based on the in situ XRD and ex situ TEM analyses, three reversible lithiation‐induced phase transitions between M1, M2, O1, and O2 phase were proposed. This was also energetically corroborated by DFT calculations. It is noteworthy that fullerenes play an important role in modulating the open‐circuit voltage of solar cells and organic molecules such as poly(3‐hexylthiophene‐2,5‐diyl) and tetrakislawsone have been used as cathodes for photo‐rechargeable Zn‐ion and LIBs.^[^ ^68^, ^69^, ^70^, ^71^ ^]^ Therefore, this work will not only enrich the studies of multi‐electron‐redox organic electrode materials for rechargeable batteries, but also shed light on the research of fullerene battery chemistry, providing new understandings beyond conventional rechargeable batteries to inspire the research of novel batteries including the emerging solar‐rechargeable batteries.

## 4 Experimental Section

### Electrode Fabrication

Commercial C_60_ powder (MREDA TECHNOLOGY, >99.50%) was used without further processing. The C_60_ powder and acetylene black were ground together in a mortar, followed by mixing them with the suspension of polyvinylidene fluoride (PVdF) as binder in N‐methyl‐2‐pyrrolidone. The mass ratio of C_60_, acetylene black, and PVdF was controlled at 60:20:20 ratio. Next, the slurry was cast uniformly onto conductive‐carbon‐coated aluminum foil as current collector and dried at 60 °C for 10 h inside a vacuum oven. The dried coated aluminum foil was punched into electrode discs of 8 mm in diameter, where the mass loading of the electrode material is ≈0.83–1.33 mg cm^−2^. Other details are presented in Supporting Information.

### Electrochemical Measurements

GDC tests were carried out at room temperature on the battery tester (NEWARE). CV tests were carried out at various scan rates at room temperature using an electrochemical workstation (CHI 660E). EIS measurements were conducted by applying a perturbation voltage of 5 mV in the frequency range from 100 kHz to 0.01 Hz using the same electrochemical workstation. Other details are included in Supporting Information.

### Materials Characterization

The morphology image of pristine C_60_ was obtained by a field emission SEM (TESCAN MIRA3, accelerating voltage: 15 kV). Powder XRD data for pristine C_60_ were collected on Bruker Advance D8 system with Cu K*α* radiation (*λ* = 1.5418 Å). TEM images were acquired using Cs‐corrected Environmental TEM operated at 80 kV. Other characterization details are provided in Supporting Information.

### Computational Method

All calculations were conducted by using the Vienna ab initio simulation package code, based on the DFT method. Details can be found in Supporting Information.

## Conflict of Interest

The authors declare no conflict of interest.

## Supporting information

Supporting Information

## Data Availability

Research data are not shared.
