# Supplementary material for: Probing Mechanistic Insights into Highly Efficient Lithium Storage of C60 Fullerene Enabled via Three‐Electron‐Redox Chemistry
Source: Adv Sci (Weinh). 2021 Jul 11;8(17):2101759. doi: 10.1002/advs.202101759 (PMC8425916; doi:10.1002/advs.202101759)
Supplement: Supplementary file 1 — Supporting Information [file ADVS-8-2101759-s001.pdf]

© 2021 Wiley-VCH GmbH

ADVANCED SCIENCE Open Access

Supporting Information

for *Adv. Sci.*, DOI: 10.1002/advs.202101759

Probing Mechanistic Insights into Highly-Efficient Lithium Storage of C60 Fullerene Enabled via Three-Electron-Redox Chemistry

*Haifa Qiu, Jing Wan, Junxian Zhang, Xin Wang, Nianji Zhang, Rouxi Chen, Yu Xia, Li Huang and Hsing-Lin Wang**

Supporting Information

Probing Mechanistic Insights into Highly-Efficient Lithium Storage of C_{60} Fullerene Enabled via Three-Electron-Redox Chemistry

Haifa Qiu,[a,b] Jing Wan,[d] Junxian Zhang,[b] Xin Wang,[e] Nianji Zhang,[b] Rouxi Chen,[b] Yu Xia,[b] Li Huang[e] and Hsing-Lin Wang* [a,b, c]

a. Shenzhen Key Laboratory of Solid State Batteries, Southern University of Science and Technology, Shenzhen 518055, China
b. Department of Materials Science and Engineering, Southern University of Science and Technology, Shenzhen, 518055, China.
c. Guangdong Provincial Key Laboratory of Energy Materials for Electric Power, Southern University of Science and Technology, Shenzhen 518055, China
d. Department of Physics, Southern University of Science and Technology, Shenzhen, 518055, China.
e. Academy for Advanced Interdisciplinary Studies, Southern University of Science and Technology, Shenzhen, 518055, China.

*Correspondence Author: Hsing-Lin Wang, E-mail: wangxl3@sustech.edu.cn

Experimental section:

1. Electrode fabrication

Commercial C_{60} powder (MREDA TECHNOLOGY, >99.50%) was used without further processing. The C_{60} powder and acetylene black were ground together in a mortar, followed by mixing them with the suspension of polyvinylidene fluoride (PVdF) as binder in *N*-methyl-2-pyrrolidone (NMP). The mass ratio of C_{60}, acetylene black and PVdF was controlled at 60:20:20. Next, the slurry was cast uniformly onto conductive-carbon-coated aluminum foil as current collector and dried at 60 °C for 10 h inside a vacuum oven. The dried coated aluminum foil was punched into electrode discs of 8mm in diameter, where the mass loading of the electrode material is ~0.83-1.33 mg/cm^2. The assembly of CR2016-type coin cells for electrochemical testing were conducted inside an argon-filled glovebox with H_2O and O_2 content below 0.1 ppm. The coin cells adopt the as-fabricated C_{60} electrode disc as cathode (working electrode) and lithium metal as anode (counter/reference electrode). A polypropylene film (Celgard) was used as the separator, and three types of electrolytes were used as the electrolyte inside cells, including EL-1, the electrolyte of 1 M $LiPF_6$ dissolved in mixed solvent of ethylene carbonate (EC), diethyl carbonate (DEC), and dimethyl carbonate (DMC) (1:1:1 in volume), EL-2, the electrolyte of 1M $LiClO_4$ in the mixture of dimethyl ether (DME) and dioxolane (DOL) (1:1 in volume) and EL-3, the electrolyte of 1M LiTFSI in DME and DOL (1:1 in volume).

2. Electrochemical measurements

Galvanostatic discharge-charge (GDC) tests were carried out in the voltage range of 1.2-3.0 V at current densities at room temperature on the battery tester (NEWARE). Cyclic voltammetry (*CV*) tests were carried out at various scan rates at room temperature using an electrochemical workstation (CHI 660E). Electrochemical impedance spectroscopy (EIS) measurements were conducted by applying a perturbation voltage of 5 mV in the frequency range from 100 kHz to 0.01 Hz using the same electrochemical workstation. Galvanostatic intermittent titration (GITT) tests were performed using the same battery tester. The GDC procedure for GITT comprises galvanostatic discharge/charge current pulses of 20 mA/g (0.2 C) for 10 minutes alternated with 2-hour open-circuit shelfing duration to reach quasi-equilibrium potentials. The apparent lithium-ion diffusion coefficients (D) of the C_{60} cathode at different charged and discharged states were derived from the

D

$\_ \; 4 \left(IV_{\mathrm{m}} \right) \left(\quad dE/dx \quad \right)$

GITT measurement via the following relation[1]:

In the formula, I stands for the applied current, V_{m} for the molar volume of C_{60} molecule, F for Faradic constant 96486 C/mol, S for the contact area between the C_{60} and electrolyte, which was calculated based on the Brunauer-Emmett-Teller (BET) surface area of C_{60} and the C_{60} mass in the electrode disc, *dE/dx* for the slope of the plot of the open circuit voltage (OCV) versus lithium content (also known as the Coulometric titration curve), $dE/d(t^{1/2})$ for the slope of the plot of the

transient operating voltage versus square root of the GDC pulse time. All electrochemical measurements were performed using coin cells.

3. Materials characterization

The morphology image of pristine C_{60} was obtained by a field emission scanning electron microscopy (SEM, TESCAN MIRA3, accelerating voltage: 15 kV). Powder X-ray diffraction (XRD) data for pristine C_{60} were collected on Bruker Advance D8 system with Cu *Kα* radiation (λ = 1.5418 Å). Transmission electron microscope (TEM) images were acquired using *Cs*-corrected Environmental TEM operated at 80 kV. The specific surface area for pristine C_{60} was characterized by N_2 isothermal adsorption/desorption test through a Micromeritics Porosimeter test station (ASAP 2460) using BET method. The *ex situ* attenuated-total-reflection infra-red (ATR-IR) spectroscopy tests were carried out on Nicolet IS10. The samples for ATR-IR tests were obtained via disassembling the coin cells at various discharged/charged states, followed by washing with DME solvent and drying and reserving under argon atomosphere before characterization. The samples for *ex situ* TEM were also obtained by cell disassembly, followed by partially dissolving the electrode materials in DME.

In situ XRD tests were performed on Rigaku Smartlab 9 kw (D/Max-2400, Cu Kα, λ = 1.54056 Å), with XRD data collected successively every 6 minutes.

In situ Raman spectroscopy tests were conducted using a confocal micro-Raman spectrometer (HORIBA *LabRAM HR Evolution*) with 532 nm excitation laser source.

Computational method

All calculations were conducted by using the Vienna ab initio simulation package (VASP) code, based on the density functional theory (DFT) method.[2] Projector augmented wave (PAW) based potentials were used to describe the interactions between valence electrons and ion cores.[3] Generalized gradient approximation (GGA) with Perdew-Burke-Ernzerhof (PBE) was used to treat the exchange-correlation interactions.[4] The energy and force convergence values were set at 10^{-5} eV and 0.01 eV/Å, respectively. The Kohn-Sham orbitals were expanded in plane waves with cut-off energy of 500 eV. The Brillouin zone integration and *k*-point sampling were performed with a Monkhorst-Pack scheme of a 2×2×2 grid for different phases of Li_xC_{60} (x = 0, 1, 2, 3) and bare FCC C_{60}.[5] The LiC_{60} O1 *Pnnm*, Li_2C_{60} M1 *C2/m* , Li_3C_{60} M2 *C2/m* , and C_{60} O2 *Pnnm* phase structures were calculated based on ICSD-75612, ICSD-55493, ICSD-94237, and ICSD-94500, respectively. In addition, DFT-D3 dispersion correction method was used to describe the van der Waals (VDW) interaction in these systems.[6] The visualization and the simulation of powder diffraction patterns of the structures were enabled using VESTA software.[7]

The simulated lithium deintercalation voltage (E_d) vs. Li/Li^+ was derived from a series of energy computations, with the entropic contribution assumed as negligible and the desolvation energy of LiTFSI, Li^+ and $TFSI^-$ disregarded, as[8]

$$E_d = \frac{E(C_{60}) + xE_b(Li) - E_{Li_xC_{60}}}{x}$$

where, x is the number of lithium ions per unit cell, $E_{Li_xC_{60}}$ is the energy of the intercalated fullerene or the fulleride, E(C_{60}) the energy of C_{60} in O2 (orthorhombic phase) at fully charged state, E_b(Li) the energy per Li atom in the bulk metal: -183.491362895 KJ/mol (equivalent to -1.90175445 eV) per Li atom.

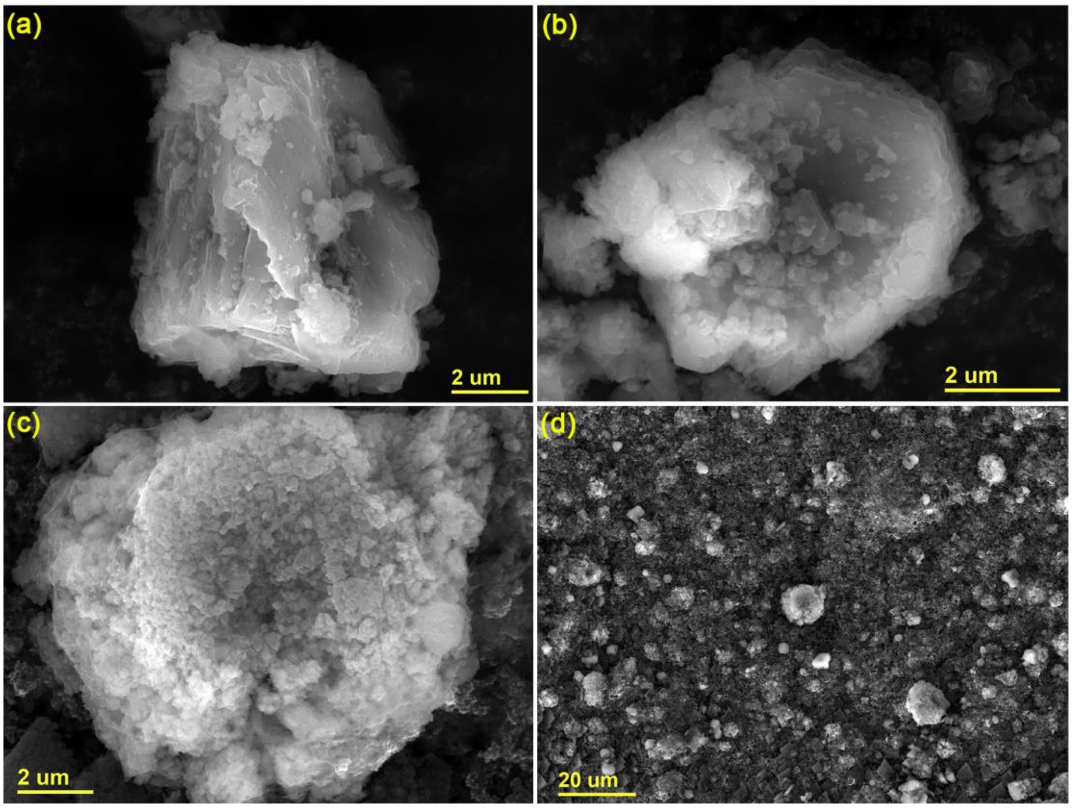

Figure S1. The morphology of (a-b) the pristine C_{60}, and (c-d) the C_{60} in electrode disc.

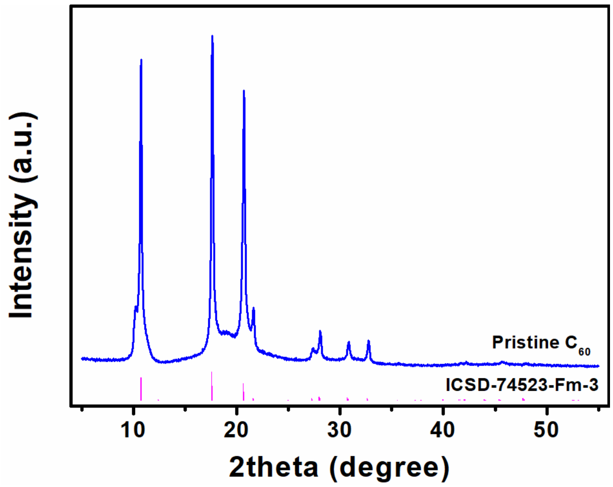

Figure S2. The powder XRD pattern of the pristine C_{60} powder.

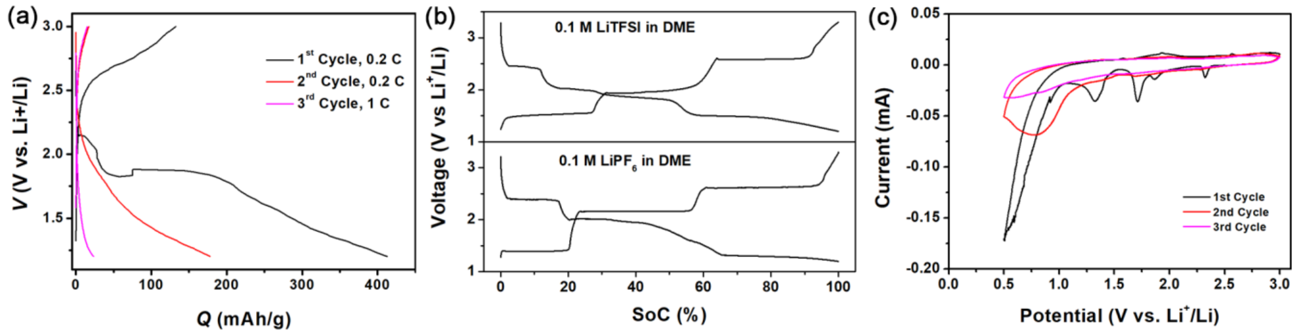

Figure S3. (a) The voltage profiles for the first three GDC cycles at various current rates for EL-1 electrolyte, (b) The voltage profiles at 0.1 C current rate for various electrolytes, (c) The *CV* curves for the first three cycles in the potential range between 0.5 and 3.0 V for EL-3 electrolyte. In Figure S3a, it was observed that the GDC voltage profiles for EL-1 show a long plateau which is largely irreversible and no obvious plateaus in following cycles. This is quite different from those for ether-based electrolytes. Figure S3b shows the C_{60} has redox reactivity in electrolyte of 0.1 M $LiPF_6$ in DME, which implies that the difference in electrolyte anion make little difference. Instead, the difference may lie more in the varied interaction between different solvent and Li ions and solvent co-intercalation tend to happen in carbonate-based electrolytes for carbon-based electrodes.[9] Figure S3c shows irreversible reduction reaction happened around 0.8 V, which led to the fast deterioration of C_{60} cathode. This suggests the suitable lower limit of cut-off discharge voltage is highly important for the cathode stability.

Table S0. A comparison in activation energy for typical electrolyte systems

Elecrolyte	Activation energy E_a/ kJ/mol	Reference
EC	104.2 (calculated)	*J. Electrochem. Soc.* **2006**, 153**,** A2192.
$LiPF_6$ in EC and DMC (1:1)	68.0	*J. Phys. Chem. C* **2007**, 111, 7411-7421.
1 M LiTFSI in DME and DOL (1:1)	51.7	*Chem. Commun.*, **2019**, 55, 13211.
1 M $LiClO_4$ in DME and DOL	38.6	*Energy Environ. Sci.*, **2019**, *12, 2741*

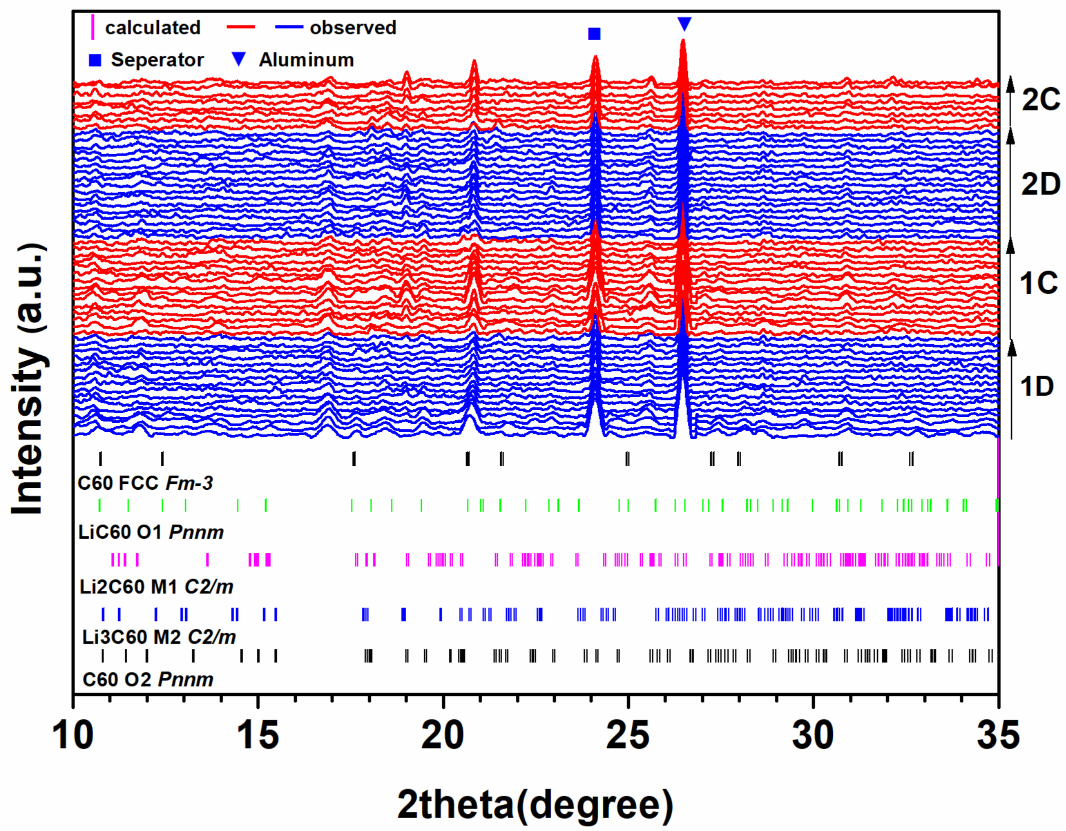

Figure S4. The *in situ* XRD patterns of the C_{60} cathode during GDC processes. It was observed there were a couple of new peaks emerging and subsiding down during GDC process. The intensities of peaks especially those at ca. 19.5° and ca. 20.8° exhibited periodic regular alteration during GDC process, which suggested the presence of reversible phase transitions.

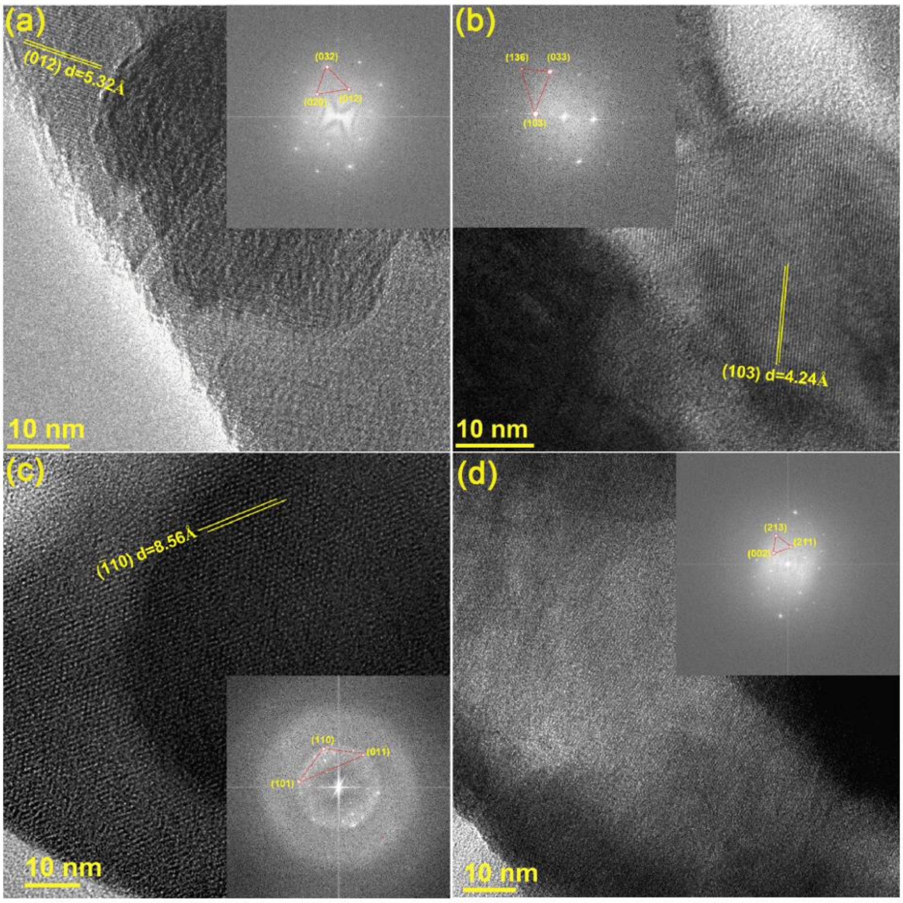

Figure S5. High resolution TEM images for C_{60} cathode samples at various discharged or charged states. TEM image for the C_{60} cathode discharged to 2.1 V (a), 1.6 V (b), 1.2 V (c), and for the C_{60} cathode charged to 3.0 V (d). Insets are corresponding images after Fast Fourier transformation (FFT) treatment.

As observed in Figure S5, the C_{60} cathode samples discharged to 2.1 V, 1.6 V, 1.2 V and the sample charged to 3.0 V are all crystalline and exhibit varied FFT patterns in the insets. For the C_{60} cathode sample discharged to 2.1 V, the d-spacings of lattice fringes and corresponding reciprocal lattice points of (020), (012), and (032) shown in the inset of Figure S5a matches the O1 phase (details are shown in Figure S4), which further confirms that the C_{60} transforms from the initial cubic phase to the O1 phase presumably in the form of LiC_{60} after the first discharge voltage plateau. Likewise, for the C_{60} cathode sample discharged to 1.6 V, as shown in Figure S5b, the spacings of specific crystal planes of (103), (033), and (136) and their reciprocal counterpart in the FFT pattern agree well with those of M1 phase, proving that the O1 phase transforms into M1 phase after the second discharge voltage plateau. Similarly, as shown in Figure S5c, for the sample discharged to 1.2 V, particular lattice spacing and reciprocal lattice points of ($\bar{1}$01), (01$\bar{1}$), and ($\bar{1}$10) consist with the M2 phase, supporting the M1 to M2 phase transformation at the third discharge voltage plateau. While for the sample fully charged to 3.0 V, as shown in Figure S5d, the spacing of crystal planes of (002), (211), and (213) and their reciprocal lattice points match the O2 phase rather than the initial FCC cubic

phase, validating the presence of irreversible structural transformation. In summary, all the TEM results are consistent with the *in situ* cXRD results.

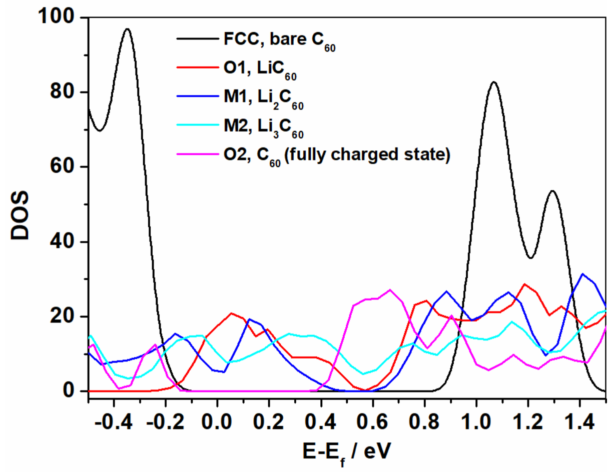

Figure S6. The density of states (DOSs) for various proposed phase structures of Li_xC_{60} (x = 0, 1, 2, 3) and bare FCC C_{60}. According to the DOS plots, the bare FCC C_{60} shows large bang gap of ca. 1 eV, while it is ca. 0.5 eV for the C_{60} at fully charged state More interestingly, the band gap disappears for all of the lithiated C_{60} phases. The DOS curves in the vicinity of the Fermi level for the M1 and M2 phases appears semimetal-like while the O1 phase is metal like, indicating that all of them possess, theoretically, intrinsic electronic conductivity. And this may result in lower charge transfer resistance, which agrees well with the results mentioned in article and shown in **Figure 4f**. Strangely, although M2 phase should be highly conductive, its corresponding R_{ct} (measured when the cell discharged to 1.2 V) is relatively high. This seems contradictory at first sight. Yet when we scrutinized the evolution of peak intensities for the peaks located at ca. 19° and 20.8° , these peaks became broaden and showed a relatively reduced intensity when the cell discharged to 1.2 V, compared with those at discharge plateaus. This reflects the lithiated fulleride Li_3C_{60} may have

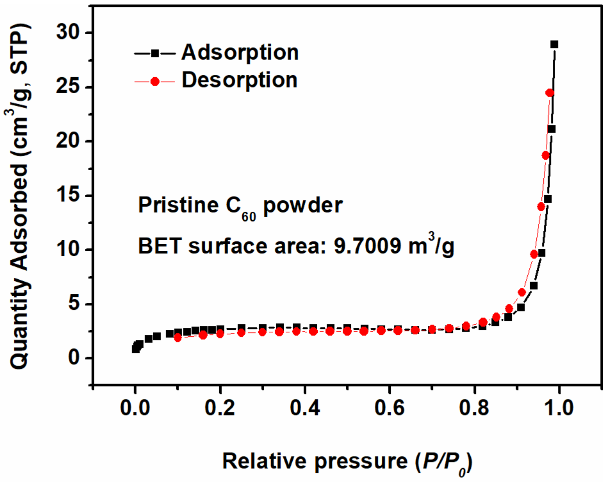

relatively lower overall crystallinity, which can also lead to increased charge transfer resistance.

Figure S7. The N_2 adsorption/desorption isotherms of the pristine C_{60} powder.

Table S1. A comparison in electrochemical performance for various metal-free organic cathode materials

Materials [a)]	Initial reversible capacity [mAh/g]	Initial Coulombic efficiency [b)]	Cycling retention [b)]	Discharge energy density [c)] [Wh/kg]	High rate capacity [mAh/g]	Rate retention	Reference
C_{60} in Li-ion battery	110 @20mA/g	91%	84% @50 cycle	158	77 @0.5A/g	64% vs 20mA/g	**Our work**
C_{60} in Mg-ion battery	39 @19uA/cm^2	76%	10% @10 cycle (75 uA/cm^2)	7.5	23 @1515 uA/cm^2	45% vs 19uA/cm^2	10
C_{60} in dual ion battery	47	35%	n/a	n/a	n/a	n/a	11
Coronene	40 @20mA/g	67%	85% @900 cycle	140	20 @0.5A/g	50% vs 20mA/g	12
Perylene	90 @20mA/g	82%	56% @1800 cycle	180	n/a	n/a	13
Triphenylene	95 @20mA/g	62%	32% @10 cycle	72	n/a	n/a	13
Conjugated copolymer: P1a	34 @1C	89%	100% @ 100 cycle	122	34 @5C	97% vs 1C	14
Conjugated copolymer: P3	28 @1C	86%	112% @100 cycle	99	26 @5C	87% vs 1C	14
4,4',4''-Tris(carbazol-9-yl)-triphenylamine (TCTA) @3.0-3.9V	28 @200mA/g	92%	39% @100 cycle	40	n/a	n/a	15
4,4',4''-Tris(carbazol-9-yl)-triphenylamine (TCTA) @3.0-4.1V	44 @200mA/g	63%	98% @100 cycle	159	n/a	n/a	15
**Thionated-naphthalene diimide, cis-2S**	120 @50mA/g	n/a	63% @400 cycle	188	60 @500mA/g	57% vs 50mA/g	16
**4,4'-(phenazine-5,10-diyl)dibenzoate anion, PZDB**	57 @123mA/g	74%	100% @200 cycle	138	48 @246mA/g	84% vs 61.5mA/g	17
**Al_2O_3-coated-Dithianon (DTN)@135.6 mA/g (0.5C)**	226 @20mA/g	56%	83% @ 1000cycle, 5C	377	210 @5C	70% vs 0.2C	18
**Polymerized Dopamine （39%）**	213 @250mA/g	92%	67% @50cycle	371	80 @10A/g	38% vs 100mA/g	19
**Polymerized Dopamine （56%）**	148 @250mA/g	94%	82% @50cycle	316	140 @10A/g	74% vs 100mA/g	19
**Polyimides@CNT**	180 @1.5A/g	90%	83% @10000 cycle	300	n/a	n/a	20
**$C_{42}H_{12}O_{12}$**	430 @0.1C	102%	52% @150cycle	506	100 @2C	29% vs 0.1C	21
**C_6O_6**	900 @20mA/g	83%	84% @100 cycle	1980	445 @500mA/g	59% vs 50mA/g	22

[a] The items in normal font refer to organic cathode materials without carbonyl group while the items in bold font refer to organic cathode materials with carbonyl group(s);
[b] Normally corresponding to the condition for initial reversible capacity. For metal-ion battery, the initial Coulombic efficiency (ICE) is calculated by the ratio of discharge capacity to its charge counterpart. While for dual ion battery, the ICE is derived from the ratio of charge capacity to its discharge counterpart.
[c] The discharge energy density after cycles is adopted, corresponding to the cycle for cycling retention. It is estimated based on the area value under the discharge voltage profile by the weight of active material.

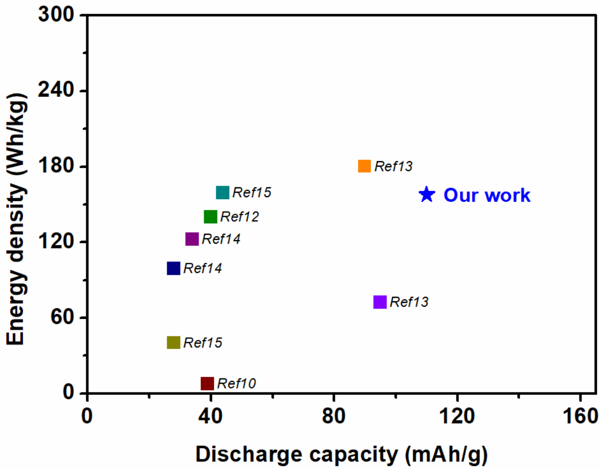

Figure S8. A comparison of battery performance for metal-free organic cathode materials without carbonyls based on the data in **Table S1**.

Table S2. A comparison in lithium ion diffusion coefficient (D_{Li^+}) of C60 and other typical electrodes as well as solid electrolyte in LIBs.

Material	D_{Li^+} / cm^2/s	Reference
C60	1.83×10^{-13}	**This work**
$LiFePO_4$	5.2×10^{-14}	*Electrochim. Acta* **2010**, *55*, 922–926
$LiNi_{0.5}Mn_{0.5}O_2$	1.41×10^{-13}	*Electrochim. Acta* **2012**, *66*, 88–93.
BQbTPL	5.30×10^{-11}	*ACS Appl. Mater. Interfaces* **2021**, *13*, 7, 9064–9073
DAAQ-TFP-COF	2.48×10^{-11}	*J. Am. Chem. Soc.* **2017**, *139*, 4258–4261.
AQ-COF	7.34×10^{-12}	*Sustainable Energy Fuels*, **2020**, *4*, 4179–4185
AQ-COF@CNTs	1.87×10^{-10}	
DMPZ	9.7×10^{-12} ~ 1.3×10^{-10}	*Angew. Chem. Int. Ed.* **2020**, *59*, 4023–4034.
Ga-LLZO (solid electrolyte)	6.2×10^{-13}	*ACS Appl. Mater. Interfaces* **2020**, *12*, 32806–32816
Al-LLZO (solid electrolyte)	6.5×10^{-15}	

Table S3. A comparison in charge transfer resistance R_{ct} of MFO cathode materials for LIBs

MFO material	R_{ct} value/Ohm	Reference
C60 fullerene	560-660	**This work**
PDA-1,<br>PDA-2	1336	*Energy Environ. Sci.*, **2017**, *10*, 205–215
PDA-4,<br>PDA-8	2051–2370	
PTCDA	>1000	*Adv. Mater.* **2020**, *32*, 2000140.
PTCDA@GDY	300-600	
PTCDA	1113	*J. Mater. Chem. A*, **2013**, *1*, 6366–6372
PTCDA@1%CNT	~1113	
PTCDA@2%CNT	602	
PTPA	600	*J. Mater. Chem. A*, **2014**, *2*, 20083–20088
PTPAn	~920	*Electrochim. Acta* **2018**, *286*, 187–194
DAAQ	746	*Sustainable Energy Fuels*, **2020**, *4*, 4179–4185
AQ-COF	736	

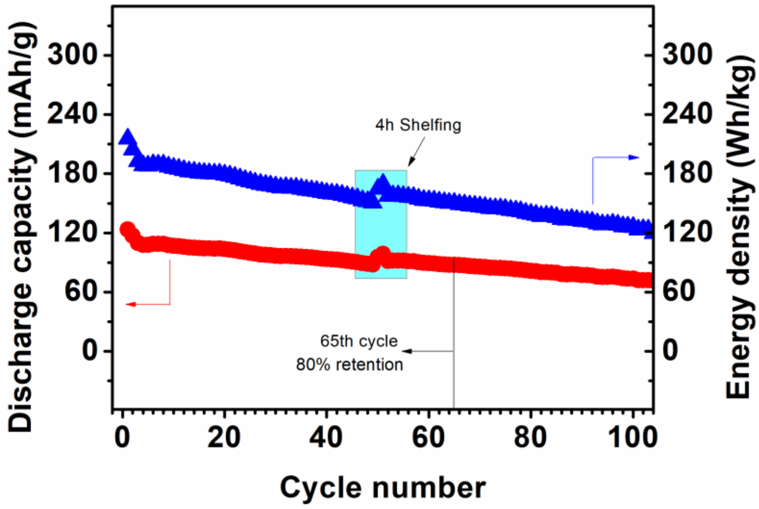

Figure S9. The cycling performance of C_{60} during longer cycles at current density of 100 mA/g. The capacity retention (relative to the 3rd cycle) is 80% after the 65th cycle and 66% after the 103rd cycle. It was found that the capacity of C60 cathode can quickly resume after short-duration shelfing time like 4 hours after 50 cycles. Even after 100 cycles, a capacity above 70 mAh/g was retained.

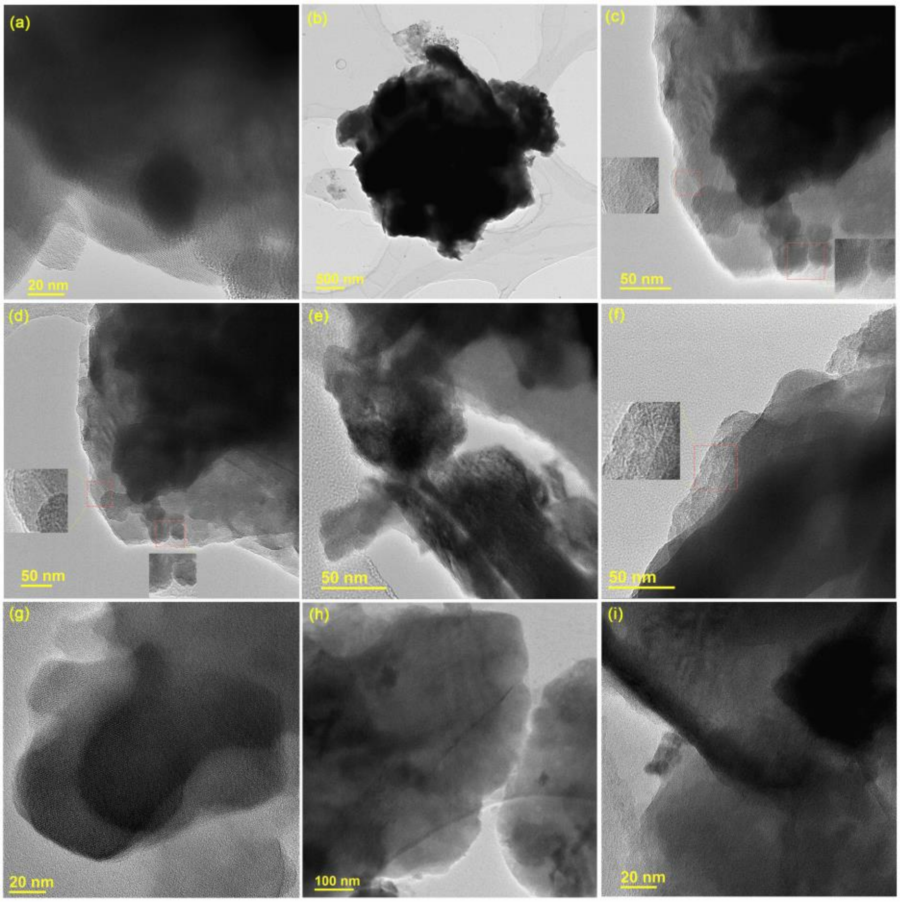

Figure S10. Morphology of C_{60} at various charge/discharge state. (a) Pristine C_{60}, (b-d) C_{60} sample discharged to 2.1V, (e-f) C_{60} sample discharged to 1.6V, (g) C_{60} sample discharged to 1.2V, (h-i) C_{60}

sample charged back to 3.0V. Figure S10b-d corresponds to Figure S5a, Figure S10e-f corresponds to Figure S5b, Figure S10g corresponds to Figure S5c, and Figure S10h-i corresponds to Figure S5d.

It can be observed that all the samples were stacked by C_{60} layer by layer, similar to the case of Figure S1b. It is noteworthy that the discharged sample is relatively sensitive to the electron beams. For example, the insets in Figure S10c shows clearly lattice fringes of lithiated C_{60} sample, while the lattice fringes became blurry in the same spot in Fig S10d after short while of TEM operation. This extrinsically made difference to the clearness of the sample. In addition, for the sample discharged to 1.6V, unlike the clear lattice fringes observed in the bulk region in Figure S5b, the onion-like lattice fringes in the interfacial region presumably imply the presence of partial polymerization at the interface as shown in the left-wing inset of Figure S10f.

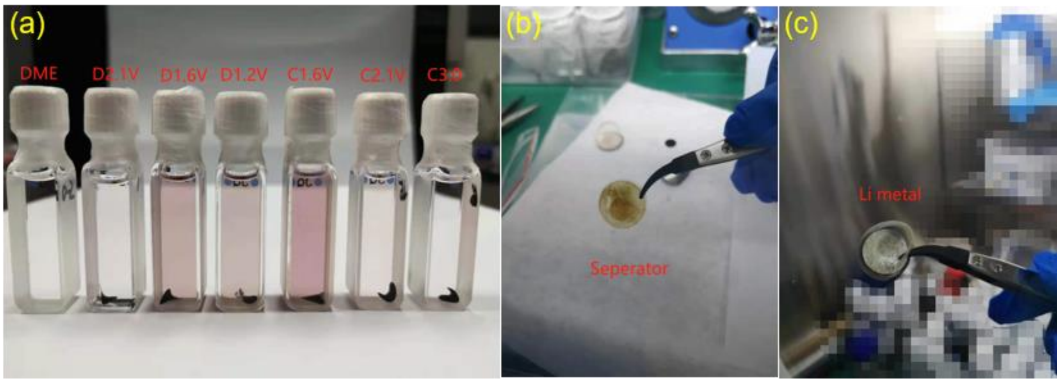

Fig. S11 The dissolution of active materials. (a) The dissolution of active materials for samples at varied charged/discharged states. (b-c) Disassembly of discharged cell.

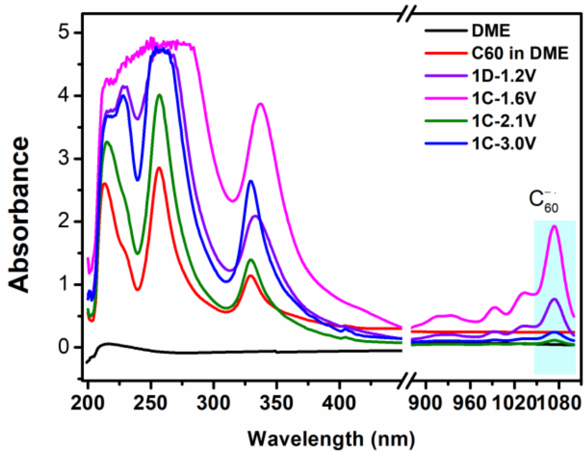

Fig S12 The UV–vis spectra for the pristine C60 and the samples at varied states of charge.

Fig S11a that the solvent is transparent for pristine electrode before discharge/discharge, and it evidently turned light brown for discharged/charged states, then it became transparent for the fully charged electrode. The results indirectly indicate the dissolution of active materials in electrolyte. As shown in Fig. S11b, the color of separator also changed and was covered by brownish species which could be the dissolved active materials. There are also dark grey spots in Li metal in Fig S11c, which can be deposited species. Most importantly, UV–vis spectroscopy was conducted to qualitatively probe the dissolution of active materials. As shown in Fig S12, the strong peak at ca. 330 nm is assigned to the excitonic transition of $H_u \rightarrow T_{1g}$ in C_{60} molecule, while the peak at around 1073 nm in near-infrared region, present in the spectra of all samples except the pristine C_{60} and DME, was reported to be characteristic of the anion radial $C_{60}^{\dot{-}}$,[23,24] which verifies the dissolution of C_{60} for all the electrodes at varied state of charge. And the change in the intensity of the characteristic peak at 1076 nm is basically consistent with the color change of solutions for various states charges in Fig S11a. Specifically, the dissolution degree was relatively lower at fully charged states yet higher at deeply discharged states.

References:

[1] Wen, C. J.; Boukamp, B. A.; Huggins, R. A.; Weppner, W. *J. Electrochem. Soc.* **1979**, *126*, 2258–2266.
[2] Kresse, G.; Hafner, *Phys. Rev. B* **1993**, *47*, 558–561.
[3] Kresse, G.; Furthmuller, *Phys. Rev. B* **1996**, *54*, 11169–11186.
[4] Perdew, J. P.; Burke, K.; Ernzerhof, M. *Phys. Rev. Lett.* **1996**, *77*, 3865–3868.
[5] Monkhorst, H. J.; Pack, J. D. *Phy. Rev. B* **1976**, *13*, 5188–5192.
[6] Grimme, S.; Antony, J.; Ehrlich, S.; Krieg, H. *J. Chem. Phys.* **2010**, *132*, 154104.
[7] Momma, K.; Izumi, F. *J. Appl. Crystallogr.* **2011**, *44*, 1272–1276.
[8] Yang, C.; Chen, J.; Ji, X.; Pollard, T. P.; Lü, X.; Sun, C.-J.; Hou, S.; Liu, Q.; Liu, C.; Qing, T.; Wang, Y.; Borodin, O.; Ren, Y.; Xu, K.; Wang, C. *Nature* **2019**, *569*, 245–250.
[9] Jiang, L.-L.; Yan, C.; Yao, Y.-X.; Cai, W.; Huang, J.-Q.; Zhang, Q. *Angew Chem., Int. Ed.* **2020**, *n/a*.
[10] Zhang, R.; Mizuno, F.; Ling, C. *Chem. Commun.* **2015**, *51*, 1108–1111.
[11] Ishihara, T.; Koga, M.; Matsumoto, H.; Yoshio, M. *Electrochem. Solid-State Lett.* **2007**, *10*, A74.
[12] Rodriguez-Perez, I. A.; Jian, Z. L.; Waldenmaier, P. K.; Palmisano, J. W.; Chandrabose, R. S.; Wang, X. F.; Lerner, M. M.; Carter, R. G.; Ji, X. L. *ACS Energy Lett.* **2016**, *1*, 719–723.
[13] Rodríguez-Pérez, I. A.; Bommier, C.; Fuller, D. D.; Leonard, D. P.; Williams, A. G.; Ji, X. *ACS Appl. Mater. Interfaces* **2018**, *10*, 43311–43315.
[14] Acker, P.; Rzesny, L.; Marchiori, C. F. N.; Araujo, C. M.; Esser, B. *Adv. Funct. Mater.* **2019**, *29*, 1906436.
[15] Zhao, C.; Chen, Z.; Wang, W.; Xiong, P.; Li, B.; Li, M.; Yang, J.; Xu, Y. *Angew. Chem., Int. Ed.* **2020**, *59*, 11992–11998.
[16] Zhang, B.; Zhang, Y.; Yang, X.; Li, G.; Zhang, S.; Zhang, Y.; Yu, D.; Liu, Z.; He, G. *Chem. Mater.* **2020**, *32*, 10575–10583.

[17] Dai, G.; He, Y.; Niu, Z.; He, P.; Zhang, C.; Zhao, Y.; Zhang, X.; Zhou, H. *Angew. Chem., Int. Ed.* **2019**, *58*, 9902–9906.
[18] Cui, C.; Ji, X.; Wang, P.-F.; Xu, G.-L.; Chen, L.; Chen, J.; Kim, H.; Ren, Y.; Chen, F.; Yang, C.; Fan, X.; Luo, C.; Amine, K.; Wang, C. *ACS Energy Lett.* **2020**, *5*, 224–231.
[19] Liu, T.; Kim, K. C.; Lee, B.; Chen, Z.; Noda, S.; Jang, S. S.; Lee, S. W. *Energy Environ. Sci.* **2017**, *10*, 205–215.
[20] Fan, X.; Wang, F.; Ji, X.; Wang, R.; Gao, T.; Hou, S.; Chen, J.; Deng, T.; Li, X.; Chen, L. J. A. C. *Angew. Chem., Int. Ed.* **2018**, *130*, 7264–7268.
[21] Huang, W. W.; Zheng, S. B.; Zhang, X. Q.; Zhou, W. J.; Xiong, W. X.; Chen, J. *Energy Storage Mater.* **2020**, *26*, 465–471.
[22] Lu, Y.; Hou, X.; Miao, L.; Li, L.; Shi, R.; Liu, L.; Chen, J. *Angew. Chem., Int. Ed.* **2019**, *58*, 7020–7024.
[23] Z. Gasyna, L. Andrews, P. N. Schatz, *J. Phys. Chem.* **1992**, *96*, 1525–1527.
[24] T. Kato, T. Kodama, T. Shida, T. Nakagawa, Y. Matsui, S. Suzuki, H. Shiromaru, K. Yamauchi, Y. Achiba, *Chem. Phys. Lett.* **1991**, *180*, 446–450.
